# Surgical Anatomy of the Gastrointestinal Tract and Its Vasculature in the Laboratory Rat

**DOI:** 10.1155/2016/2632368

**Published:** 2015-12-27

**Authors:** Katarína Vdoviaková, Eva Petrovová, Marcela Maloveská, Lenka Krešáková, Jana Teleky, Mario Zefanias Joao Elias, Darina Petrášová

**Affiliations:** ^1^Department of Anatomy, Histology and Physiology, University of Veterinary Medicine and Pharmacy in Kosice, Komenského 73, 041 81 Kosice, Slovakia; ^2^Faculty of Veterinary Medicine, Eduardo Mondlane University, P.O. Box 254, 0001 Maputo, Mozambique; ^3^Laboratory of Research Biomodels, Faculty of Medicine, Pavol Jozef Šafárik University in Kosice, Trieda SNP 1, 04011 Kosice, Slovakia

## Abstract

The aim of this study was to describe and illustrate the morphology of the stomach, liver, intestine, and their vasculature to support the planning of surgical therapeutic methods in abdominal cavity. On adult Wistar rats corrosion casts were prepared from the arterial system and Duracryl Dental and PUR SP were used as a casting medium and was performed macroscopic anatomical dissection of the stomach, liver, and intestine was performed. The rat stomach was a large, semilunar shaped sac with composite lining. On the stomach was very marked fundus, which formed a blind sac (*saccus cecus*). The rat liver was divided into six lobes, but without gall bladder. Intestine of the rat was simple, but cecum had a shape as a stomach. The following variations were observed in the origin of the cranial mesenteric artery. On the corrosion cast specimens we noticed the presence of the anastomosis between middle colic artery (*a. colica media*) and left colic artery (*a. colica sinistra*). We investigated the second anastomosis between middle colic artery and left colic artery. The results of this study reveal that the functional anatomical relationship between the rat stomach, liver and intestine is important for the development of surgical research in human and veterinary medicine.

## 1. Introduction

The laboratory animals are suitable subjects for many modern experimental and biomedical research including metabolic and immunological studies, tumor and cancer investigation, anatomical, physiological, and biochemical research, and experimental transplantation. Many structures or organs of the body of laboratory animals were studied by many authors, for instance, the mouse [1, 2], hamster [3, 4], and rabbit [5, 6], but some details have not yet been examined. Frequently, the laboratory mammals are also used as animal models for veterinary and human research. The knowledge of anatomical variations is more important for experimental investigation and surgical practice. The investigation of anatomy, comprising the morphology of the vessels in laboratory animals, is nearly united with ischemia and transplantation of the organs.

Nowadays, the laboratory rat is one of the most popular experimental models for the research, because it is easy to handle and inexpensive. The laboratory rat is by far the most used animal model in transplantation of liver and intestine [7, 8] and gastrointestinal tract diseases studies. Monchik and Russell made in 1971 the first experimental transplantation of the intestine in laboratory rats [9]. Compared to humans, the laboratory rats have similar anatomical structure of the body organs.

The aim of this study was to describe and illustrate the morphology of the gastrointestinal tract and its vasculature to support the planning of surgical therapeutic methods in abdominal cavity. The topography of the gastrointestinal organs and variations of the vascularisation are more important in the methods of experimental ischemia and the transplantations.

## 2. Material and Methods

The experiment was carried out on 20 laboratory rats (*Rattus norvegicus f. domestica*) aged one year, breed Wistar of both sexes (10 females, 10 males), and weighing approximately 350–520 g in the standard breeding condition. Animals were obtained from accredited Laboratory of Research Biomodels, University of P. J. Safarik in Kosice. The experiment on rats was performed with approval of the Ethics Committee of the University of Veterinary Medicine and Pharmacy in Kosice and State Veterinary and Food Institute in Bratislava (number SK P 12004) followed by Slovakian protocols for ethical standards for the use of laboratory animals. The first group of rats (10 animals) was used for the corrosion casts of the arteries and the second group of rats (10 animals) was used for dissection of the gastrointestinal organs.

### 2.1. The Preparing of the Corrosion Casts Specimens of the Arterial System

Anaesthesia of animals was induced by intraperitoneal injection of sodium thiopental (50 mg/kg, Thiopental Valeant, Valeant Czech Pharma, Czech Republic). Under total anaesthesia we dissected the left ventricle of the heart. We implemented a cannula into the aorta through the left ventricle while the cannula was supported by ligature. A portion of the venous system must be opened to ensure a good distribution of the perfusion medium. The right auricular appendage served this purpose. Vessels were perfused with isotonic saline 0,9% physiological solution at a low flow rate (about 10 mL/min) for 30 s through the left cardiac ventricle. An improved method for the washing out of clotted blood from the vessels was achieved by the addition of 0,05% NaOH (Mikrochem, Slovakia) into the perfusion medium. The perfusion pressure was approximately 200–250 mm Hg (2,6–3,25 m H_2_O). The success of the perfusion was indicated by the uniform fading of the tissues seen during the procedure. We mixed the injection media in stechiometric rates. The corrosion casts were prepared with Duracryl Dental resin (Spofa, Dental, Czech Republic) and PUR SP resin (Ústav polymérov, SAV, Slovakia). Suitable colour tone was achieved by addition of 2-3 drops of red (oil, red paint 0). After a proper mixing of all components we applied this mass into the arterial system through the left ventricle of the heart. After vascular casting with the resin is complete, it (and the animal) must not be manipulated for 30 min, after which the casts are submersed in water at a temperature ranging from 40°C to 60°C for a period between 30 min and 24 h for full polymerization of resin [10]. The maceration of the soft tissues was carried out in 2–4% solution of KOH (Mikrochem, Slovakia) at 60–70°C. The maceration took approximately 2-3 days. Prior to the outset of the drying process, the corroded specimens were submersed in water and dried at room temperature. The results were listed in percentages.

### 2.2. The Macroscopic Anatomical Dissection of the Gastrointestinal Organs

In the second group of rats under total anaesthesia, macroscopic anatomical dissection of the stomach, liver, and intestine was performed and features were compared to humans. The abdominal cavity was opened by the mid-laparotomy, through the abdominal wall in the midline (*linea alba*) from the caudal end of the sternum (*processus xiphoideus*) to the pubic bone (*pecten ossis pubis*). The abdominal wall was cut on both sides, cranially along the last rib and caudally along the inguinal region. A stereostatic microscope (Leica M 320) was used for anatomical dissection, and pictures were taken with a digital camera adapted to the microscope. The latest edition of the Veterinary Anatomic Nomenclature was consulted throughout this study [11].

## 3. Results

### 3.1. Stomach Anatomy

**Figure 1:**
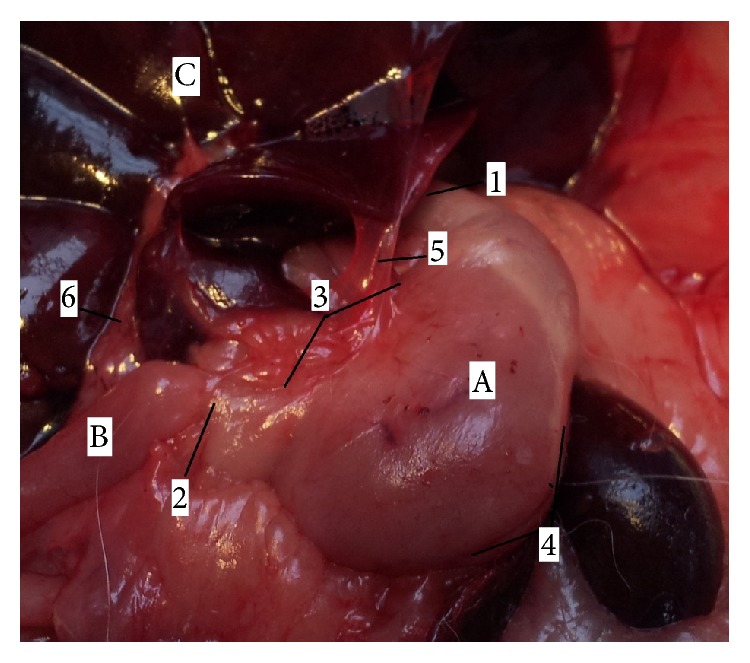
Morphology of rat stomach (*facies parietalis*). 1:* pars cardiaca*, 2:* pars pylorica*, 3:* curvatura ventriculi minor*, 4:* curvatura ventriculi major*, 5:* lig. hepatogastricum*, 6:* ductus hepaticus communis*, A:* ventriculus* (*facies parietalis*), B:* duodenum*, and C:* facies visceralis hepatis*.

**Figure 2:**
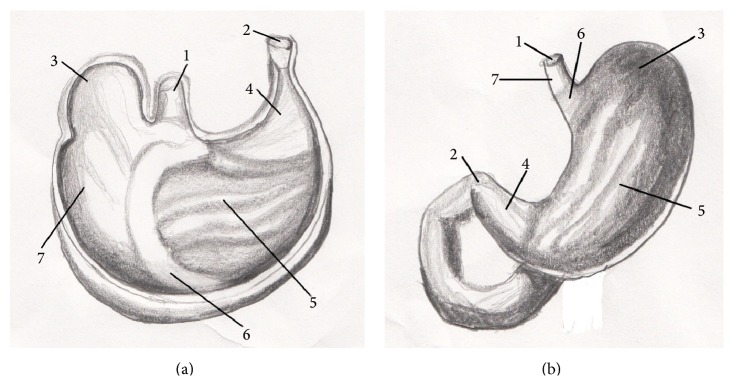
The comparative anatomy of gastric mucous membrane in rat and human. 1:* pars cardiaca*, 2:* pars pylorica*, 3:* fundus ventriculi*, 4:* glandulae pyloricae*, 5:* glandulae gastricae propriae*, 6:* glandulae cardiacae*, 7:* pars nonglandularis*, (a) rat stomach (*facies visceralis*), and (b) human stomach (*paries anterior*).

The stomach (*ventriculus*) of rat was situated in the left part of the abdominal cavity, at the level of the last thoracic and first lumbar vertebrae, dorsally to the liver and it was directed transversally. The rat stomach was a large, semilunar shaped sac and weighed between 3,90 and 8,50 g. The stomach tissue represented approximately 1,8% of the total body weight. The left part of the stomach was cardiac part (*pars cardiaca*) and the right was pyloric part (*pars pylorica*). We described two surfaces on the rat stomach. The cranial, parietal surface (*facies parietalis*) was in contact with diaphragm and left abdominal wall. The part of the parietal surface was covered by left lobe of the liver. The caudal, visceral surface (*facies visceralis*) was attached to the intestine. The omentum majus separated jejunum (*jejunum*) and cecum (*cecum*) from the greater omentum (*facies visceralis*) of the stomach. These two surfaces were fused in greater and lesser curvature (*curvature major* and* minor*). The greater curvature was directed caudoventrally. The esophagus entered in the middle of the lesser curvature, which was directed craniodorsally. The stomach had a very marked fundus (*fundus ventriculi*), which formed a distinct craniodorsal blind ventricular sac (*saccus cecus ventriculi*) on the left side, near the cardiac part (Figure 1). Between stomach and abdominal wall adipose pad was situated, which was contributed according to sex into mesorchium (*mesorchium*), mesovarium (*mesovarium*), and mesometrium (*mesometrium*). The rat stomach was joined to visceral surface of the liver by the hepatogastric ligament (*lig. hepatogastricum*). The spleen bordered on the greater curvature of the stomach. These two organs were fused by the gastrosplenic ligament (*lig. gastrolienale*). The tunica mucosa was divided into two parts. The right glandular part (*pars glandularis*) was opaque, muscular, thick walled, and reddish containing the fundic and pyloric regions. This part included gastric glands (cardiac, proper gastric, and pyloric). The cardiac glands were found behind the nonglandular part. There was the narrow strip of cardiac glands (*glandulae cardiacae*) next to the line of transition of the mucous membrane. The pyloric glands were presented only in a zone around the right part of the stomach, around the pylorus (*pylorus*). The surface between these two groups of the glands was covered by the proper gastric glands, in the part of the area of the body of stomach (*corpus ventriculi*). The left, nonglandular part (*pars nonglandularis*) of the stomach was translucent and thin walled (Figure 2). This mucous membrane presented the continuation of the nonglandular mucous membrane of esophagus. This portion was used in the storage and digestion of food.

### 3.2. Liver Anatomy

**Figure 3:**
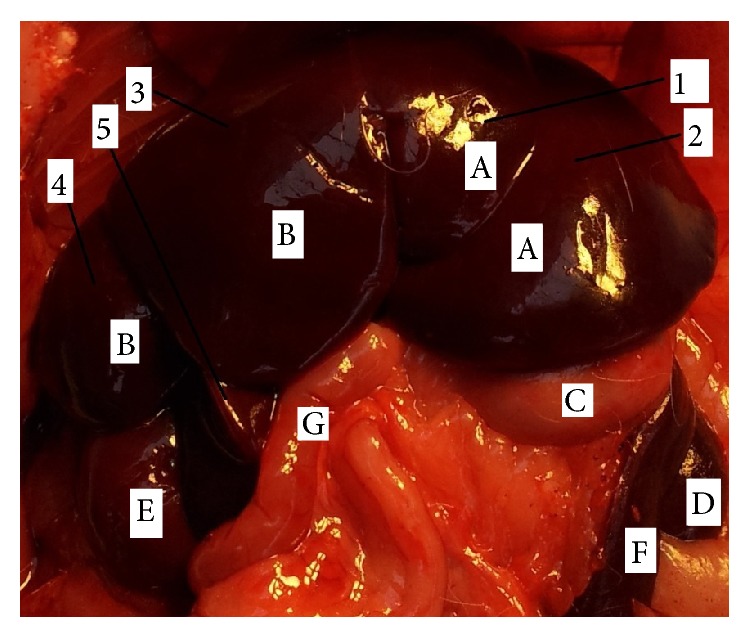
Morphology of rat liver (*facies diaphragmatica*). 1:* lobus hepatis sinister medialis*, 2:* lobus hepatis sinister lateralis*, 3:* lobus hepatis dexter medialis*, 4:* lobus hepatis dexter lateralis*, 5:* lobus quadratus*, A:* lobus hepatis sinister*, B:* lobus hepatis dexter*, C:* ventriculus*, D:* ren dexter*, E:* ren sinister*, F:* lien*, and G:* intestinum*.

**Figure 4:**
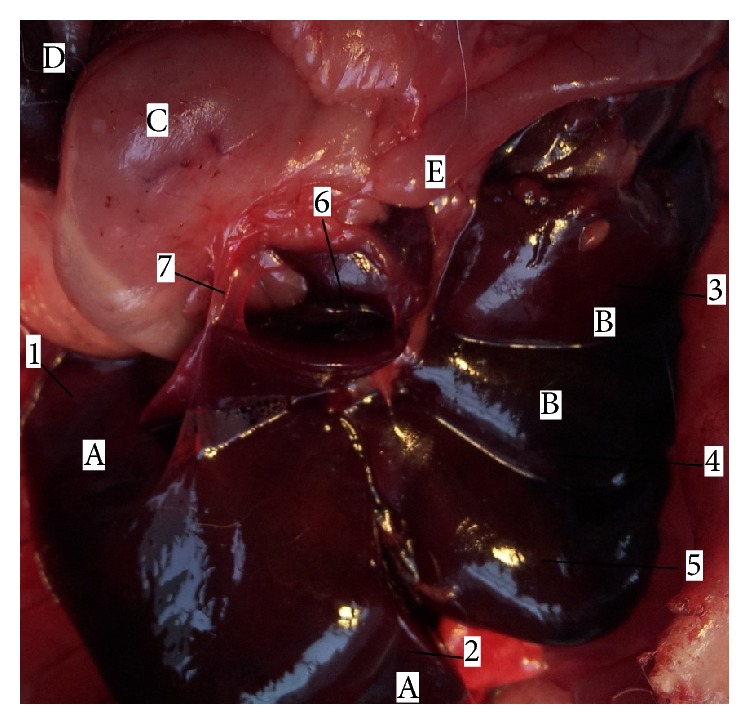
Morphology of rat liver (*facies visceralis*). 1:* lobus hepatis sinister lateralis*, 2:* lobus hepatis sinister medialis*, 3:* lobus hepatis dexter lateralis*, 4:* lobus hepatis dexter medialis*, 5:* lobus quadratus*, 6:* lobus caudatus*, 7:* lig. hepatogastricum*, A:* lobus hepatis sinister*, B:* lobus hepatis dexter*, C:* ventriculus*, D:* lien*, and E:* intestinum*.

The position of rat liver (*hepar*) was in the right side of the abdominal cavity; it was attached to the diaphragm within the rib cage. The liver extended alongside the abdominal wall ventrally beyond the ribs. The rat liver was multilobulated; liver tissue represented approximately 5% of the total body weight and it weighed approximately 13,8 g. The liver had generally two surfaces. The diaphragmatic (Figure 3), convex surface (*facies diaphragmatica*) was in contact with diaphragm and right abdominal wall. This surface was covered by the peritoneum (*peritoneum*). The falciform ligament (*lig. falciforme*) was a thin peritoneal fold and it was attached to the convex surface of the diaphragm and the caudal surface of the right abdominal wall. The visceral (Figure 4), concave surface (*facies visceralis*) was in relation to the guts (stomach, duodenum, right colic flexure, the pancreas, the right kidney, and suprarenal gland). The diaphragmatic and visceral surfaces were fused in four margins. On dorsal margin (*margo dorsalis*) of the liver was situated caudal vena cava (*v. cava caudalis*) in groove for this vein (*sulcus venae cavae*). This vein received segmental hepatic veins (*vv. hepaticae*) from the liver tissue. Dorsally to the caudal vena cava coronary ligament was situated (*lig.* coronarium) which was divided into two branches. The right branch continued into the right triangular ligament (*ligamentum triangulare dextrum*) and the left branch continued into the left triangular ligament (*ligamentum triangulare sinistrum*). On the ventral margin (*margo ventralis*) was hepatic teres ligament of the liver (*ligamentum teres hepatis*) was embedded into deep fissure, which was presented between left medial lobe and quadrate lobe. Ligament of the liver (*ligamentum teres hepatis*) formed the main continuation of the falciform ligament and it presented the obliterated rest of umbilical vein (*v. umbilicalis*). Between the liver and other organs (duodenum, stomach) were ligaments, which formed the lesser omentum (*omentum minus*). There were hepatoduodenal and hepatogastric ligaments (*ligamentum hepatoduodenale *and* hepatogastricum*). On the right and left side of the liver were the right and left margins (*margo dexter *et sinister), which bear deep fissures between the lobes. On the visceral surface was the transverse fissure of the liver (*porta hepatis*). The transverse fissure of the liver (*porta hepatis*) was the opening where entered portal vein, hepatic artery and nerves and came out hepatic duct. The laboratory rats had these six lobes of the liver: left medial, lateral and right medial, lateral lobe, caudate and quadrate lobe (*lobus hepatis sinister medialis*,* lateralis* and* lobus hepatis dexter medialis*,* lateralis*,* lobus caudatus*, and* quadratus*). Between hepatic lobes were presented deep fissures. The caudate lobe was divided into caudate and papillar process (*processus caudatus* and* papillaris*). The pointed caudate process arose from the visceral surface and it protruded dorsally, to the right side of the liver. The caudate process met the right kidney. Papillary process (*processus papillaris*) extended across the* curvatura ventriculi major* and it was in contact with the* facies visceralis* of the stomach. The gall bladder (*vesica fellea*) was absent. All hepatic ducts were fused and formed common hepatic duct (*ductus hepaticus communis*), which led to the duodenum. The common hepatic duct was situated ventrally and to the right of the portal vein (*vena portae*).

### 3.3. Intestine Anatomy

**Figure 5:**
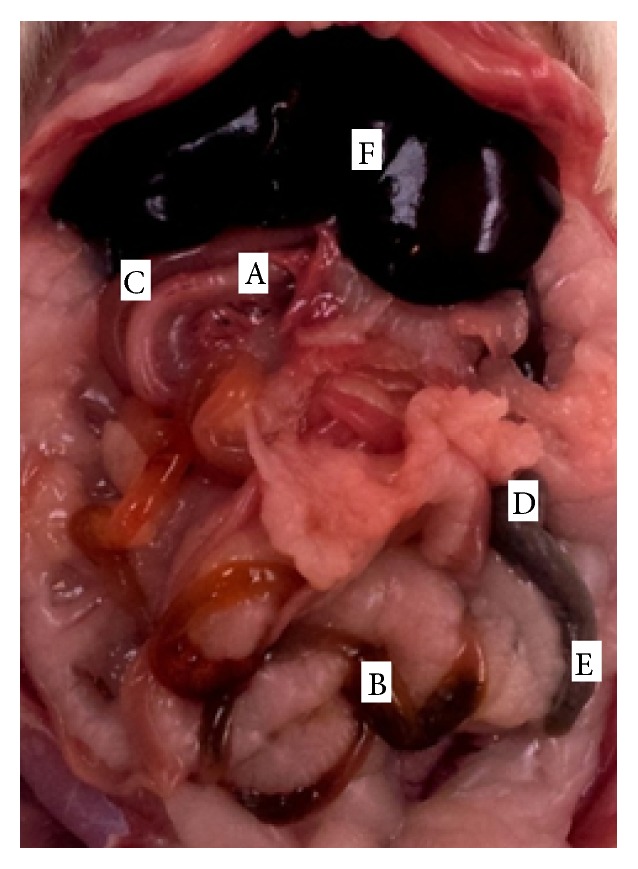
Abdominal cavity with intestine in rat, ventral view. A.* duodenum*, B.* jejunum*, C.* colon*, D.* cecum*, E.* processus vermiformis*, and F.* hepar*.

The small intestine (*intestinum tenue*) of the laboratory rat arose from pyloric part of the stomach (*pars pylorica ventriculi*). The pyloric part was situated in the right side to the median plane. The first part of the small intestine, duodenum (*duodenum*), arose from the stomach. The length of duodenum was approximately 95–100 mm (Table 3). Cranial part of duodenum (*pars cranialis duodeni*) is a part, which was situated near the visceral surface of the liver and right abdominal wall. Descending part of duodenum (*pars descendens duodeni*) continued by the right abdominal wall to the right kidney. Between cranial duodenal part and descending part of duodenum was the first flexure, cranial duodenal flexure (*flexura duodeni cranialis*). The descending part of the duodenum turned to the cranial direction, as an ascending part of duodenum (*pars ascendens duodeni*) in this position. Between the descending and ascending part of duodenum was presented the caudal duodenal flexure (*flexura duodeni caudalis*). The ascending part of the duodenum continued to the median plane to the jejunum (*jejunum*). The other flexure was between the last part of duodenum and jejunum and it was duodenojejunal flexure (*flexura duodenojejunalis*). This part of small intestine filled the right part of the abdominal cavity.* Jejunum* formed loops and it filled the right part of the abdominal cavity ventrally and it passed fluently to the ileum (*ileum*). The jejunum measured 890–1300 mm (Table 3). At the level, where ileum entered to the cecum, enlargement was presented,* sacculus rotundus*.* Sacculus rotundus* was formed by lymphatic tissue. The length of the ileum was 20–30 mm (Table 3). The opening of the ileum into the cecum was enclosed to the beginning of the colon (*colon*). The cecum was on the right caudal side of the abdominal cavity. The cecum had base (*basis*), body (*corpus*), and an apical part (*apex*). From the apex continued* processus vermiformis*, which was the last part of the* cecum*. The cecum had the long mesentery which allowed for different positional variation. Behind the* cecum*, which measured 45–65 mm (Table 3), followed the* colon*. The first part was ascending colon (*colon ascendens*); it led cranially to the thoracic cavity. From the left to the right side ran tansverse colon (*colon transversum*), the second part of the colon. On the right side of the abdominal cavity continued descending colon (*colon descendens*) (Figure 5). The length of the colon was 95–100 mm (Table 3) and rectum measured approximately 75 mm (Table 3). The rectum ran as a straight tube through the pelvis and ended just below the root of the anus. Behind rectum were anal canal (*canalis analis*) and anus (*anus*).

### 3.4. The Division of the* A. Coeliaca*

The results of our study indicated that the stomach, liver, and intestine in a laboratory rat are supplied by the three main arteries, which are the branches of the abdominal aorta. These arteries are celiac artery (*a. coeliaca*), cranial mesenteric artery (*a. mesenterica cranialis*), and caudal mesenteric artery (*a. mesenterica caudalis*).

**Figure 6:**
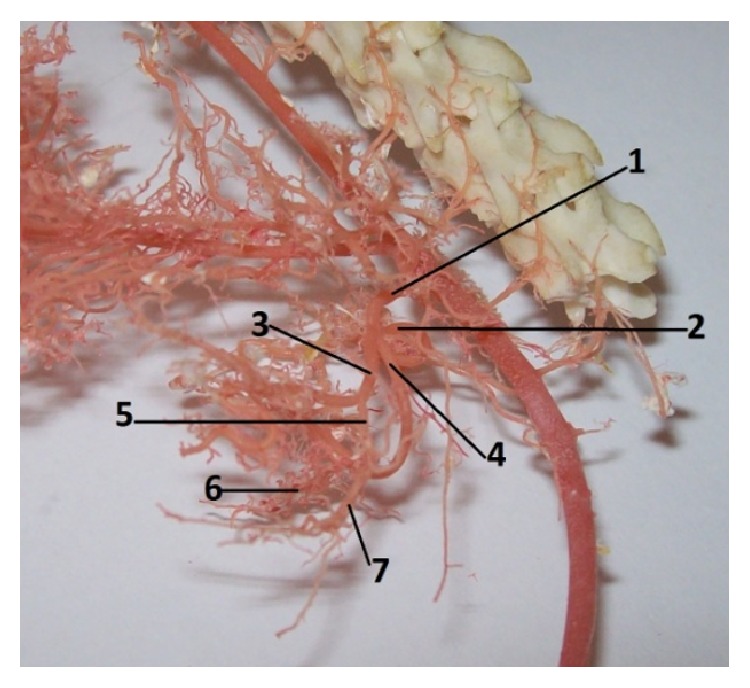
The division of the coeliac artery in rat. 1:* a. celiaca*, 2:* a. gastrica sinistra*, 3:* a. hepatica*, 4:* a. lienalis*, 5:* a. gastroduodenalis*, 6:* a. gastroepiploica dextra*, and 7:* a. pancreaticoduodenalis cranialis* (casting medium Duracryl Dental).

*Arteria coeliaca* was the first visceral branch, which left the ventral wall of the abdominal aorta (Figure 6). It was the short, unpaired trunk, which arose at the level of the third lumbar vertebra. This artery supplied stomach, spleen, liver, pancreas, and cranial part of the duodenum. The coeliac artery was divided into three main branches: splenic artery (*a. lienalis*), left gastric artery (*a. gastrica sinistra*), and hepatic artery (*a. hepatica*).

Splenic artery (*arteria lienalis*) continued after the origin on the left side, by the cranial border of the left pancreatic lobe. There was* arteria lienalis* devided into many pancreatic branches (*rr. pancreatici*) and than continued ventral to the centre of the splenic hilus. The splenic artery gave the common trunk for the splenic branches (*rr. lienales*) of the spleen. Our results indicated that* rr. lienales *were presented in numbers 5, 6, and 8 (5 in 20%, 6 in 30%, and 8 in 50%). Splenic artery then continued as the left gastroepiploic artery (*a. gastroepiploica sinistra*) on the greater curvature of the stomach. This left gastroepiploic artery was divided into short gastric arteries (*aa. gastricae breves*), which supplied the fundic region of the greater curvature of the stomach. These short gastric arteries were visible on gastric surfaces.

Left gastric artery (*arteria gastrica sinistra*) originated directly from the coeliac artery in all cases. The left gastric artery directed on the lesser curvature of the stomach in the region of the insertion of the gastric mesentery (*mesogastrium*). Along this course it gave off to the parietal branches (*rr. parietales*) and* viscerales* to the surfaces of the stomach. These short gastric branches anastomosed each other.

The last branch of the celiac artery was* a. hepatica*. It turned to the liver and gave rise to the hepatic branches, the* rr. pancreatici*, the right gastric artery (*a. gastrica dextra*), and the gastroduodenal artery (*a. gastroduodenalis*). The last branch was the parent artery of the cranial pancreaticoduodenal artery (*a. pancreaticoduodenalis cranialis*) and the right gastroepiploic artery (*a. gastroepiploica dextra*). Right gastric artery (*a. gastrica dextra*) directed on the lesser curvature of the stomach; it supplied the pyloric region. This artery followed the lesser curvature of stomach (*curvatura ventriculi minor*) and there it anastomosed with the left gastric artery. Right gastric artery gave off parietal and visceral branches to both gastric surfaces. Gastroduodenal artery (*a. gastroduodenalis*) was the common part for the greater curvature of the stomach and for the first part of duodenum. Right gastroepiploic artery was the terminal limb of the gastroduodenal artery, which was divided into the short gastric arteries for parietal and visceral surfaces of the stomach. Right gastroepiploic artery and* sinistra* anastomosed each other. Cranial pancreaticoduodenal artery was one of the branches of the gastroduodenal artery. This artery directed in the mesoduodenum (*mesoduodenum*) along the cranial and descending parts of the duodenum.

### 3.5. The Division of the Cranial Mesenteric Artery

Our results indicated that cranial mesenteric artery (*a. mesenterica cranialis*) was the thickest branch of the abdominal aorta. It was the unpaired second visceral branch from the ventral wall of the abdominal aorta, caudal to the celiac artery. This artery is more important during embryonic development, because the gut twists around this artery. The cranial mesenteric artery supplied all parts of digestive system which were attached to this mesentery and which participate in the embryonic rotation. The following variations were observed in the origin of the cranial mesenteric artery. In 9% of the corrosion cast specimens, the cranial mesenteric artery (*a. mesenterica cranialis*) originated from the abdominal aorta cranial to the origin of the right renal artery (*a. renalis dextra*) (Figure 10), and in 37% a common trunk originated from the abdominal aorta for the cranial mesenteric artery and the right renal artery (*a. renalis dextra*) (Figure 9). In 39% the* a. mesenterica cranialis* originated from abdominal aorta (*aorta abdominalis*) caudal to the right renal artery (Figure 8). Cranial mesenteric artery was divided into these branches: middle colic artery (*a. colica media*), caudal pancreaticoduodenal artery (*a. pancreaticoduodenalis caudalis*), right colic artery (*a. colica dextra*), jejunal arteries (*aa. jejunales*), and ileocecocolic artery (*a. ileocecocolica*).

The results of our study indicated that* a. colica media* was the first branch, running caudal from its origin. It supplied blood to the transverse colon and cranial portion of the descending colon. In 46% it branched off individually from the cranial mesenteric artery and in 37% as a common trunk with the right colic artery.

Inferior pancreaticoduodenal artery (*arteria pancreaticoduodenalis caudalis*) left the cranial mesentery artery in caudal direction and coursed in the mesentery of the ascending part of duodenum to the caudal duodenal flexure. This artery originated ventral to the right colic artery in laboratory rat. Inferior pancreaticoduodenal artery was divided into pancreatic branches (*rr. pancreatici*) for pancreas and duodenal branches (*rr. duodenales*) for* duodenum*. The following variations were observed in the origin of this artery. In 46% the caudal pancreaticoduodenal artery was originated cranially from the right colic artery and in 37% after the right colic artery. On the corrosion cast specimens, we noticed the presence of the anastomosis between inferior pancreaticoduodenal artery and artery superior pancreaticoduodenal (*a. pancreaticoduodenalis cranialis*).

The third branch of the cranial mesenteric artery in laboratory rat was the right colic artery. This artery ran towards the caudal part of the ascending colon. In 37% right colic artery originated from the cranial mesenteric artery as a common trunk with the middle colic artery before the origin of the inferior pancreaticoduodenal artery. In 46% right colic artery branched off independently after the origin of inferior pancreaticoduodenal artery.

Jejunal arteries originated from the cranial mesenteric artery in higher number. These arteries supplied blood to the jejunum and cranial part of ileum. Jejunal arteries ran in the mesojejunum (*mesojejunum*). On the corrosion cast specimens, we noticed the presence of a jejunal arcade and a jejunal trunk. Jejunal trunks were the common parts for the origin of the jejunal arteries from the cranial mesentery artery. Jejunal arcades were presented on the intestinal wall and they were the terminal parts of the jejunal arteries.

*Arteria ileocecocolica* arose from the cranial mesenteric artery; it directed caudoventrally; it coursed to the cecocolic junction. The principal continuation of the cranial mesenteric artery in laboratory rat was the* a. ileocecocolica*, which was divided into the colic branch (*r. colicus*) for ascending colon, ileal artery (*a. ilealis*) for ileum, and cecal artery (*a. cecalis*) for* cecum*. The presence of the* a. ileocecocolica* together with its branches was observed in 46%. Cranial mesenteric artery in 37% continued as the ileocecal artery (*a. ileocecalis*), which is bifurcated into the cecal artery (*a. cecalis*) and ileal artery and colic branch branched off independently.

### 3.6. The Division of the Caudal Mesenteric Artery

Caudal mesenteric artery (*arteria mesenterica caudalis*) was the third unpaired visceral branch to spring from the ventral wall of the abdominal aorta. This artery was a thinner branch than the cranial mesenteric artery. Caudal mesenteric artery was divided into left colic artery (*a. colica sinistra*) and cranial rectal artery (*a. rectalis cranialis*). This artery supplied blood to the caudal part of the descending colon and the rectum (Figure 11).

Our results described anastomosis between cranial pancreaticoduodenal artery and caudal pancreaticoduodenal artery. We investigated the second anastomosis between middle colic artery and left colic artery (Figure 12).

## 4. Discussion

The detailed description of the rat organs of the digestive system and their vasculature is decisive for ischemia and transplantations in this model of laboratory animals. Many previous publications and descriptions have mentioned only partial results of anatomy of these organs. We have presented integrated knowledge about a rat stomach, liver, intestine, and vasculature, which is more important for experimental ischemia and transplantations. Many authors described microstructures of these organs [13, 14]. The anatomical relationship between the human and rat digestive organs is still undefined (Tables 1, 2, and 4). The functional anatomy of the digestive organs in the rat is considered similar to humans. The rats are suitable for determining the mechanism of drug absorption and bioavailability values from powder or solution formulations [14].

The classic morphological books describe the human stomach as the most dilated part of the digestive system, beneath the diaphragm in the left hypochondriac and epigastric region of the abdominal cavity [15, 16]. Its shape and topography are closely associated with organogenesis. Some developmental abnormality of the stomach, as well as abnormality of vessels and nerves, may influence stomach morphology [17, 18]. The empty stomach has a typical, cylindrical shape with a well-formed anterior and posterior wall and lesser and greater curvature as well as fundus, cardia, body, and pylorus [19]. In distended one, the anterior wall increases the area attached to the abdominal wall. During inspiration stomach is displaced downward. Abnormal fluid accumulation in the pleural or peritoneal cavity may change the stomach shape as well [16].

**Figure 7:**
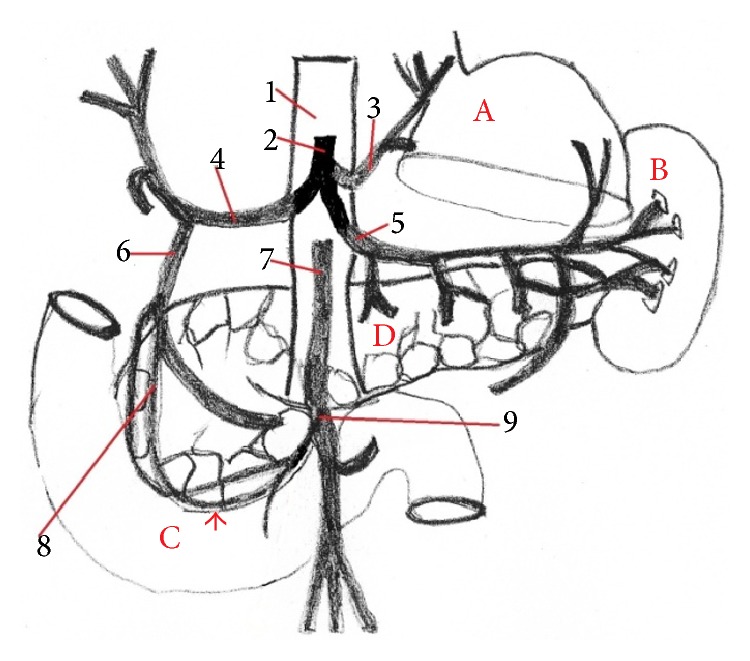
The division of the celiac trunk and the presence of the anastomosis between* a. supraduodenalis superior* and* a. pancreaticoduodenalis inferior* in human (anastomosis,* arrow*) (scheme). 1:* aorta abdominalis*, 2:* truncus ceoliacus*, 3:* a. gastrica sinistra*, 4:* a. hepatica communis*, 5:* a. splenica*, 6:* a. gastroduodenalis*, 7:* a. mesenterica superior*, 8:* a. supraduodenalis superior*, 9:* a. pancreaticoduodenalis inferior*, A: liver, B: spleen, C: duodenum, and D: pancreas.

**Figure 8:**
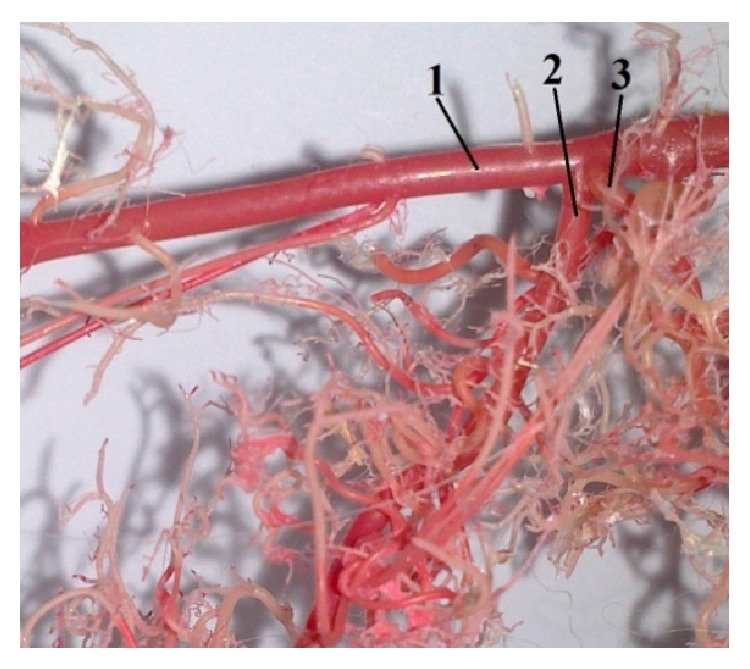
Photograph demonstrating the origin of* a. mesenterica cranialis* caudal to the* a. renalis dextra*. 1:* aorta abdominalis*, 2:* a. mesenterica cranialis*, and 3:* a. renalis dextra* (casting medium Duracryl Dental).

**Figure 9:**
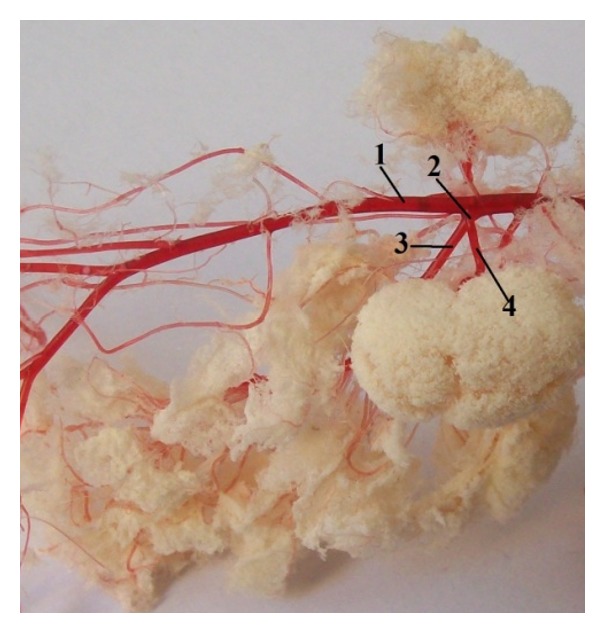
Photograph showing the common trunk of the* a. mesenterica cranialis* with the* a. renalis dextra*. 1:* aorta abdominalis*, 2: a common trunk, 3:* a. mesenterica cranialis*, and 4:* a. renalis dextra* (casting medium PUR SP).

**Figure 10:**
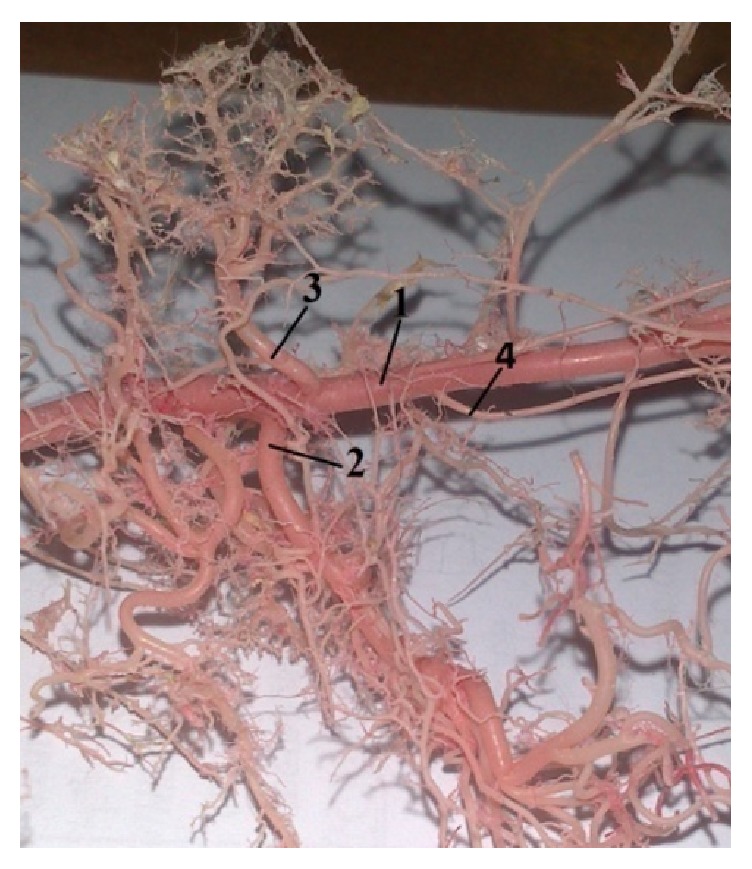
Photograph pointing out the origin of the* a. mesenterica cranial* is cranial to the* a. renalis dextra*. 1:* aorta abdominalis*, 2:* a. mesenterica cranialis*, 3:* a. renalis dextra*, and 4:* a. testicularis dextra* (casting medium Duracryl Dental).

**Figure 11:**
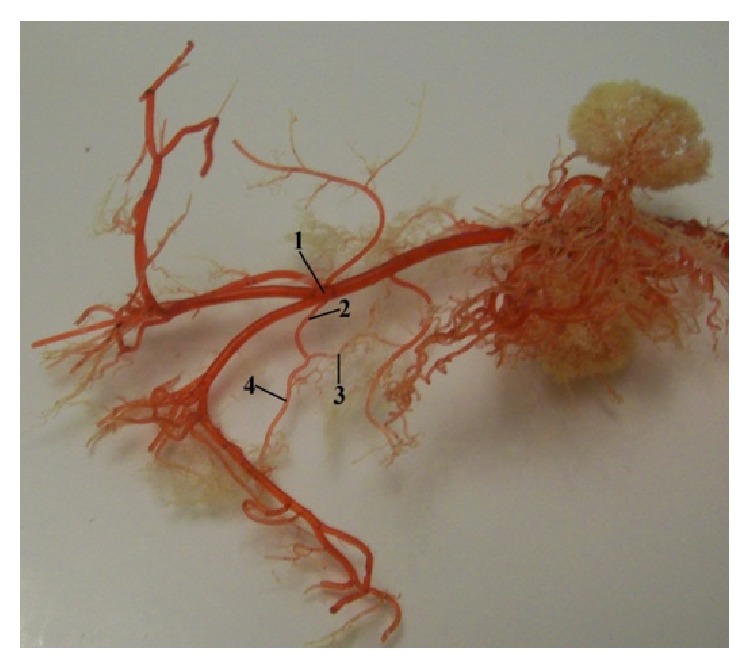
Photograph focusing the division of the* a. mesenterica caudalis*. 1:* aorta abdominalis*, 2:* a. mesenterica caudalis*, 3:* a. colica sinistra*, and 4:* a. rectalis cranialis* (casting medium PUR SP).

The general description of the human stomach [20] indicates that the cardiac part of the stomach is situated on the left side at the level of the tenth thoracic vertebra. Near the cardiac part is* fundus ventriculi*, which is simple and is filled by air. At the opposite end of the stomach is pyloric part, which has a very strong band of the smooth muscle, pyloric sphincter. In the human there is only the glandular type of the stomach and it is lined with cardiac, gastric, and pyloric mucosa (Figure 2). From domestic mammals only carnivores have the same gastric mucous membrane as a human [21]. Some species do not possess a glandular stomach and in that case the oesophageal mucosa becomes continuous with that of the small intestine [22]. Stomach is supplied by vessels of the short celiac trunk (Figure 7). The lesser curvature supplied primarily the left gastric artery which arose from celiac trunk. The right gastric artery was usually a small vessel that provided branches to the first part of duodenum and the pylorus. The right and left gastroepiploic arteries arose from the gastroduodenal and splenic arteries, respectively. They from an arcade along the greater curvature, the right provided blood to the antrum, and the left supplied the lower portion of the fundus. The short gastric arteries that arose from the splenic artery were small and relatively insignificant in terms of the amount of blood that they delivered to the most proximal portion of the body of the stomach [23]. The anatomy of the rat stomach is greatly influenced by adaptation, nature of food, body size, and shape. The division and description of the rat stomach are not uniform. The rat stomach is divided into the forestomach (pars proventricularis) and glandular stomach (corpus or pars glandularis) (Figure 2). The forestomach occupies about three-fifths of the stomach area. The glandular stomach is the other part and is divided into the fundus and pylorus [24]. The morphology of the rodent stomach is different from the stomach of other laboratory animals [25, 26]. Functionally, the forestomach serves as a storage organ [27]. The glandular stomach is functionally similar to that of the other laboratory animals [28]. The nomenclature is not the same. Some authors describe the rat stomach glandular and nonglandular parts, which are separated by the limiting ridge [29]. The rat stomach is supplied by long celiac trunk [30, 31]. The origin, division, and course of the celiac trunk (Figure 6) and its branches are similar to human, but the area of supplying of single arteries is different. The experiment of the blood supply to the gastric mucosa in rats showed these results. The left gastric artery was manifest at two sites, the greater curvature, the pylorus, and the first part of duodenum. The right gastric artery was very small and terminated by supplying the duodenum. The second largest artery after the left gastric artery was the right gastroepiploic artery which supplied most of the greater curvature. The fundus received a few small branches from the splenic artery [32].

The morphology of the rat liver has been considered different from humans [33]. The general description of the liver in human segmentation by Couinaud divided the human liver into eight lobes and this presents the basis of hepatic resections [34]. Three hepatic veins divide the liver into four parts (right lateral part, right paramedian part, left paramedian part, and left lateral part). Each part receives a portal branch which bifurcates and drains the blood from each lobe [35]. The nomenclature of the liver in rats is not identical [36–38]. Most authors divided the rat liver into these four lobes: left lateral lobe, medial lobe (left and right medial), caudate lobe (anterior or inferior, posterior, or superior), and right lobe. Popesko et al. divided the rat liver into six lobes: right hepatic lobe (lateral and medial part), left hepatic lobe (lateral and medial lobe), caudate lobe (caudate and papillary process), and quadrate lobe [39] (Figures 3 and 4). Lorente et al. divided the rat liver into two parts: superior and inferior liver and six sectors: caudate process, caudate lobe, right lateral lobe, right segment of the right medial lobe, middle, and left segment of the right medial lobe and left medial lobe; the last sector is the left lateral lobe [33]. In the study of Kogure et al., it was shown that the liver of the rat is the same as that of the humans [36]. Each lobe of the rat liver has its own arteries. These unrivalled considerations of the rat liver morphology make resections of different extents simply and highly reproducible and of low price when performing experiments [40]. Different from humans, other mammals, the rat liver does not have a gallbladder, though some mammals, like the horse, deer, or some birds, like the pigeon, do not have it either [41].

The functional anatomy of the intestine in the rat has been considered similar to humans. The length of the human intestine and its parts is not uniform.

**Figure 12:**
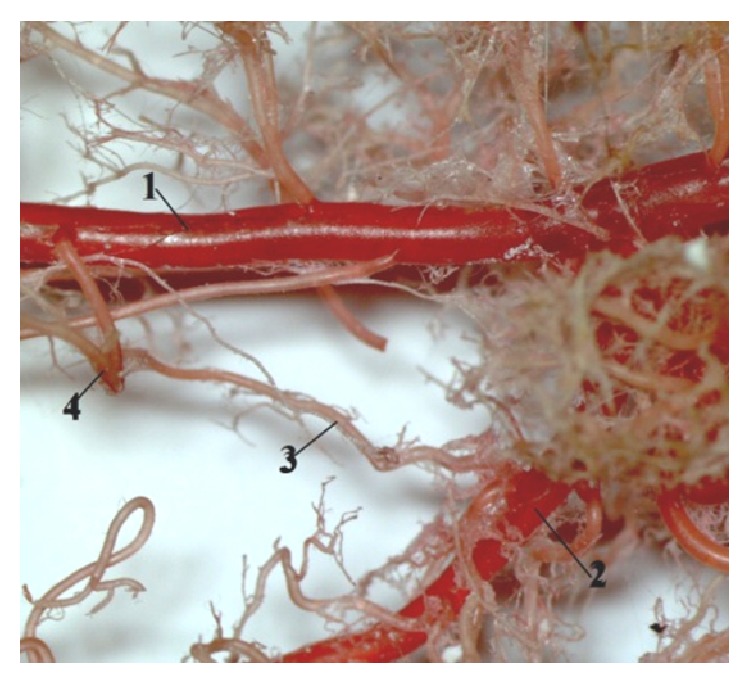
The presence of the anastomosis between* a. colica sinistra* and* a. colica media*. 1:* aorta abdominalis*, 2:* a. mesenterica cranialis*, 3:* a. colica media*, and 4:* a. colica sinistra* (casting medium Duracryl Dental).

**Figure 13:**
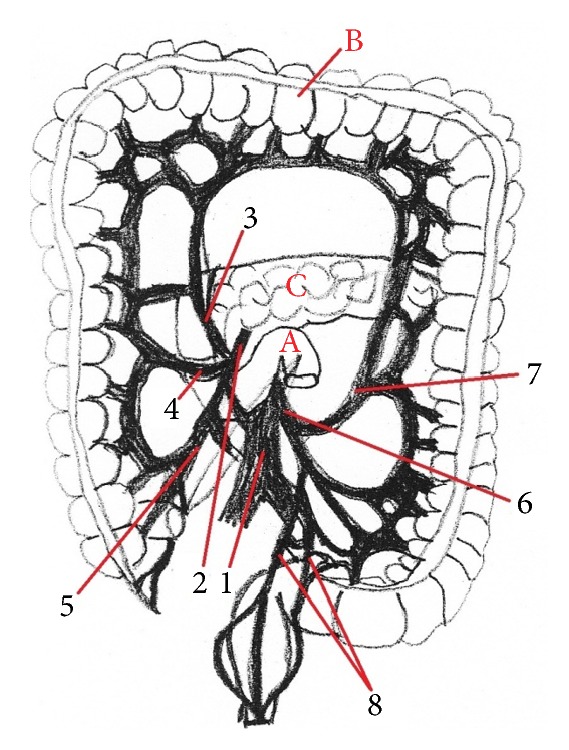
The division of the a.* mesenterica superior* in human. 1:* aorta abdominalis*, 2:* a. mesenterica inferior*, 3:* a. colica media*, 4:* a. colica dextra*, 5:* a. ileocolica*, 6:* a. mesenterica inferior*, 7:* a. colica sinistra*, 8:* aa. rectales superiores*, A:* flexura duodenojejunalis*, B:* colon*, and C:* pancreas*.

The small intestine of human is from the morphophysiological aspect the main organ of digestion in the body. This part of intestine is divided into duodenum, jejunum, and ileum. The length of the small intestine in a healthy human is approximately 5,5–6,4 m (Table 4). The minimum length of the intestine, which is needed for maintenance of the absorption, is 50–70 cm, while the full function of the large intestine is carried on. Diseases of small intestine and their vasculature form acute or chronic ischemia of intestine. The effect of these disorders is in many cases the resection of the intestinal part. It is mainly the part of jejunum and* ileum*. If you take off the intestine, there will be problems with malabsorption and maldigestion and other failures of digestion of the base of the nutriments. If these cases are not treated, the disease can eventually cause multiple organ failure [42]. The anatomical nomenclature of the intestinal supply in humans and rats is different. The rat small intestine is supplied by cranial mesenteric artery [30, 31] and by superior mesenteric artery in human [43]. The main division of this artery is the same. Some authors described in humans the anastomosis between a. ileocolica and a. colica dextra. In 5% of cases this anastomosis was absent [44]. The second anastomosis was between* a. supraduodenalis superior* and* a. pancreaticoduodenalis inferior* (Figure 13). In rats anastomosis was described between cranial pancreaticoduodenal artery and caudal pancreaticoduodenal artery (Figure 12). Our results of this anastomosis agreed with other authors [30, 45].

The large intestine is the part which is more important for absorption of water, electrolytes, and vitamins. Humans have a poorly defined cecum, which is only continuous with the colon [14]. But the position of the appendix of the cecum is very different in human. The cecum of pig is more complex than humans. In pig cecum, we can describe three longitudinal bands and three orders of sacs or haustra [21]. The rat cecum is as large as rat stomach.

The human colon played a major role in the absorption of water, salt (Na^+^, Cl^−^), and other minerals. This part of human intestine consists of the ascending, transverse, descending, and sigmoid sections. All parts of colon in human are sacculated. The sacculation of the colon in human is similar to pigs [14]. The rat colon is not sacculated, and it is so simple. Our results from the rat colon are in agreement with other authors [12, 31, 39]. The supplying of the large intestine in human and in rats is the same. Only the nomenclature of this artery is different. We called this artery* arteria mesenterica inferior* in human [43] and caudal mesenteric artery in rats [30, 31]. Some authors described anastomosis between left colic artery and middle colic artery. This anastomosis was present in 32%, and it was absent in 7%. More authors described in research the presence of meandering mesenteric artery, arc of Riolan [46]. This artery was an additional pathway between the superior and inferior mesenteric artery in human [47].

The blood supplying of the gastrointestinal tract is more important for surgical treatment in acute or chronic mesenteric ischemia or organ transplantation in human.

## 5. Conclusions

In summary, the rat is the most extensively used experimental animal model in veterinary and human surgical research of the abdominal cavity, intestinal transplantation, or the study of medicaments absorption. The progress of the surgical therapeutic methods (treatment of ischemic injury of stomach, liver, intestine, surgical resection, the congenital disorders, Crohn's disease, transplantation) and the study of stomach, liver, and intestine morphology, function, and diseases depend on new knowledge of these organs and their vasculature. We investigated the functional anatomy of the stomach, liver, and intestine and their vasculature of the laboratory rat and compared it with the human morphology. Compared to humans, the laboratory rats have similar anatomical structure of the stomach, liver, and intestine; therefore, nowadays, the laboratory rat is one of the most popular models for research of anatomical, physiological, and biochemical relations in the digestive system. Nevertheless, the relationship (function, anatomy) between them is not defined.

## Figures and Tables

**Table 1:** Morphological comparison of the rat and human stomach.

Rat stomach	Human stomach
Semilunar shaped sac	Pear-shaped sac
*Fundus ventriculi* forms blind ventricular sac	Only simple *fundus ventriculi *
The mucous membrane is divided into glandular part and nonglandular part	The mucous membrane is simple and consists of glandular part
Weight 1,8% of the total body weight	Weight 6,2% of the total body weight
Long celiac trunk	Short celiac trunk

**Table 2:** Morphological comparison of the rat and human liver.

Rat liver	Human liver
Six lobes of the liver	No clear morphological division of the lobes

Dividing of caudate lobe is obvious	No clear subdivision of the caudate lobe

Weight 5% of the total body weight	Weight 2,5% of the total body weight

Gall bladder is absent	Gall bladder is present

**Table 3:** Length and diameters of parts of rat intestinal tract [12].

	Length (mm)	Diameter (mm)
Duodenum	95–100	2,5–3
Jejunum	900–1350	4-5
Ileum	25–35	3–5
Cecum	50–70	10
Colon	90–110	10–3
Rectum	80	3–10

**Table 4:** Morphological comparison of the rat and human intestine.

Rat intestine	Human intestine
Length 1745 mm	Length 6-7 m
Small intestine 1485 mm	Small intestine 5,5–6,4 m
Large intestine 260 mm	Large intestine 1,5 m
